# Direct aortic TAVI via anterior right mini-thoracotomy using 32 mm myval for pure aortic regurgitation

**DOI:** 10.1186/s13019-024-02982-7

**Published:** 2024-08-30

**Authors:** Firas Aljanadi, Graham McNeilly, Ganesh Manoharan, Andrew McNiece, Reuben Jeganathan

**Affiliations:** 1 Department of Cardiothoracic surgery, Royal Victoria Hospital, Belfast, UK; 2 Department of Cardiology, Royal Victoria Hospital, Belfast, UK

**Keywords:** Aortic surgery, TAVI, Aortic regurgitation, Dilated aortic annulus, Aortic valve replacement

## Abstract

**Background:**

Aortic regurgitation with dilated annulus presents a technical challenge for conventional transcatheter aortic valve implantation (TAVI) procedures.

**Case presentation:**

We report a case of an 84-year-old frail patient with a history of breathlessness found to have severe aortic regurgitation and moderately impaired left ventricular systolic function. The patient underwent a successful TAVI procedure using the XL-Myval 32 mm transcatheter heart valve (THV) via an anterior right mini-thoracotomy with a direct aortic approach. The patient recovered well post-operatively with good hemodynamic resolution.

**Conclusions:**

This first in human case highlights the efficacy and potential of applying innovative approaches, such as the new sizes of Myval THV and direct aortic access via anterior right mini thoracotomy, in addressing challenging anatomical variations in TAVI procedures with good outcome.

**Supplementary Information:**

The online version contains supplementary material available at 10.1186/s13019-024-02982-7.

## Background

Transcatheter Aortic Valve Implantation (TAVI) has become a widely accepted intervention, particularly in aortic stenosis cases. However, its application in aortic regurgitation (AR) remains less common, presenting challenges, especially when dealing with a significantly enlarged aortic annuli.[1]

This case report presents a significant milestone as the first documented case of inserting a size 32 mm MyVal THV for pure AR via anterior right mini-thoracotomy (ART), showing the feasibility and effectiveness of this novel technique in addressing AR with larger annulus sizes.

## Case presentation

An 84-year-old frail man was admitted electively for TAVI with symptomatic severe AR with NYHAIII symptoms. He is an ex-smoker with a history of COPD, symptomatic PVD, and awaiting urgent TEVAR for a large abdominal aortic aneurysm (AAA). Trans-thoracic echocardiography showed severe AR, dilated LVEDD (65 mm), and moderately impaired LV function (Video 1).Coronary angiogram showed non-obstructive coronary artery disease.

**Fig. 1:**
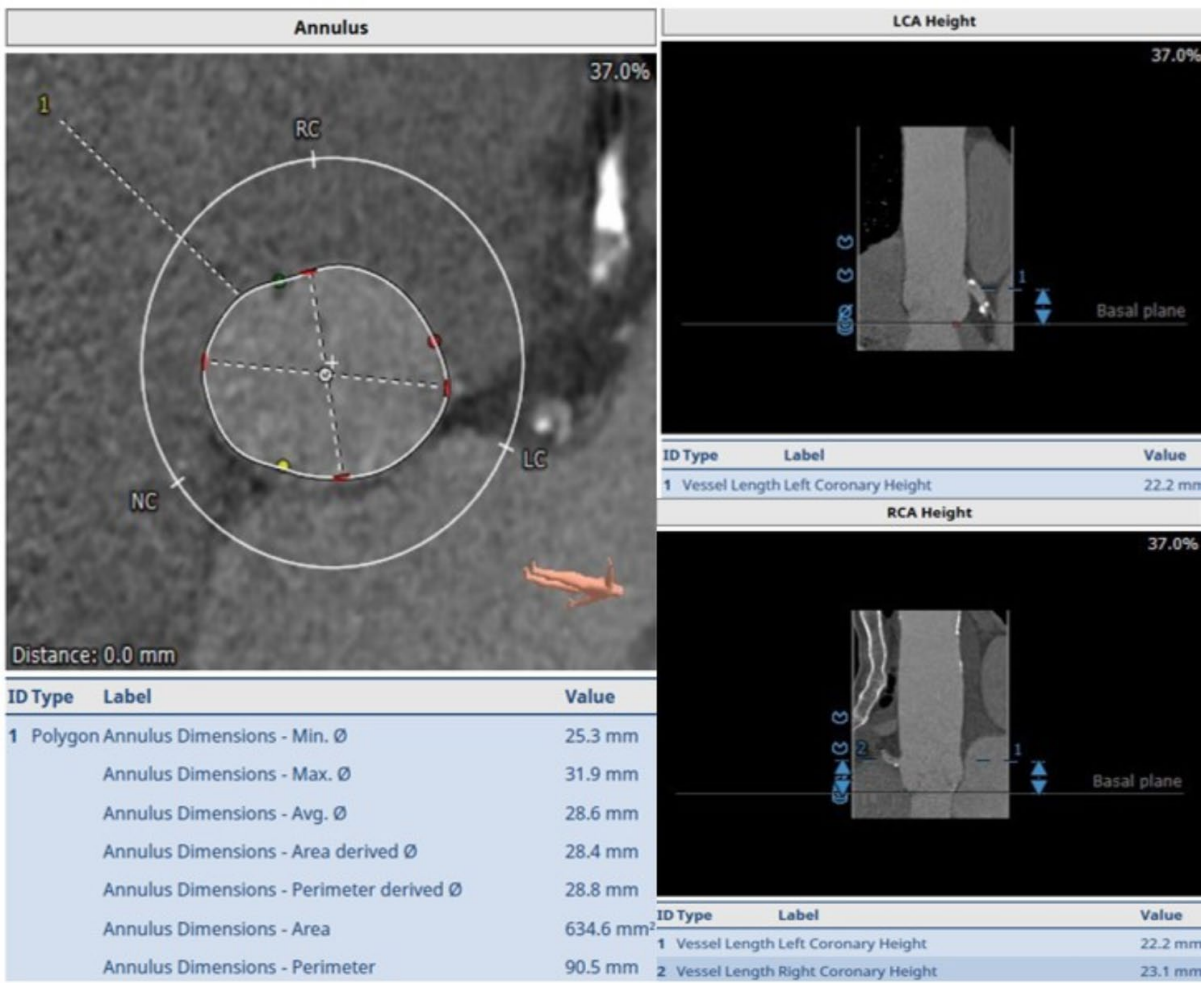
Annulus measurements on CT scan preoperatively

A cardiac CT showed a large aortic annulus (perimeter 90.5 mm, area 635mm^2^) (Fig. 1) with no calcification and a saccular infrarenal aortic aneurysm measuring 52 mm. There may have been a previous contained rupture. He was discussed by the HEART team and the consensus was for alternative access TAVI.

**Fig. 2:**
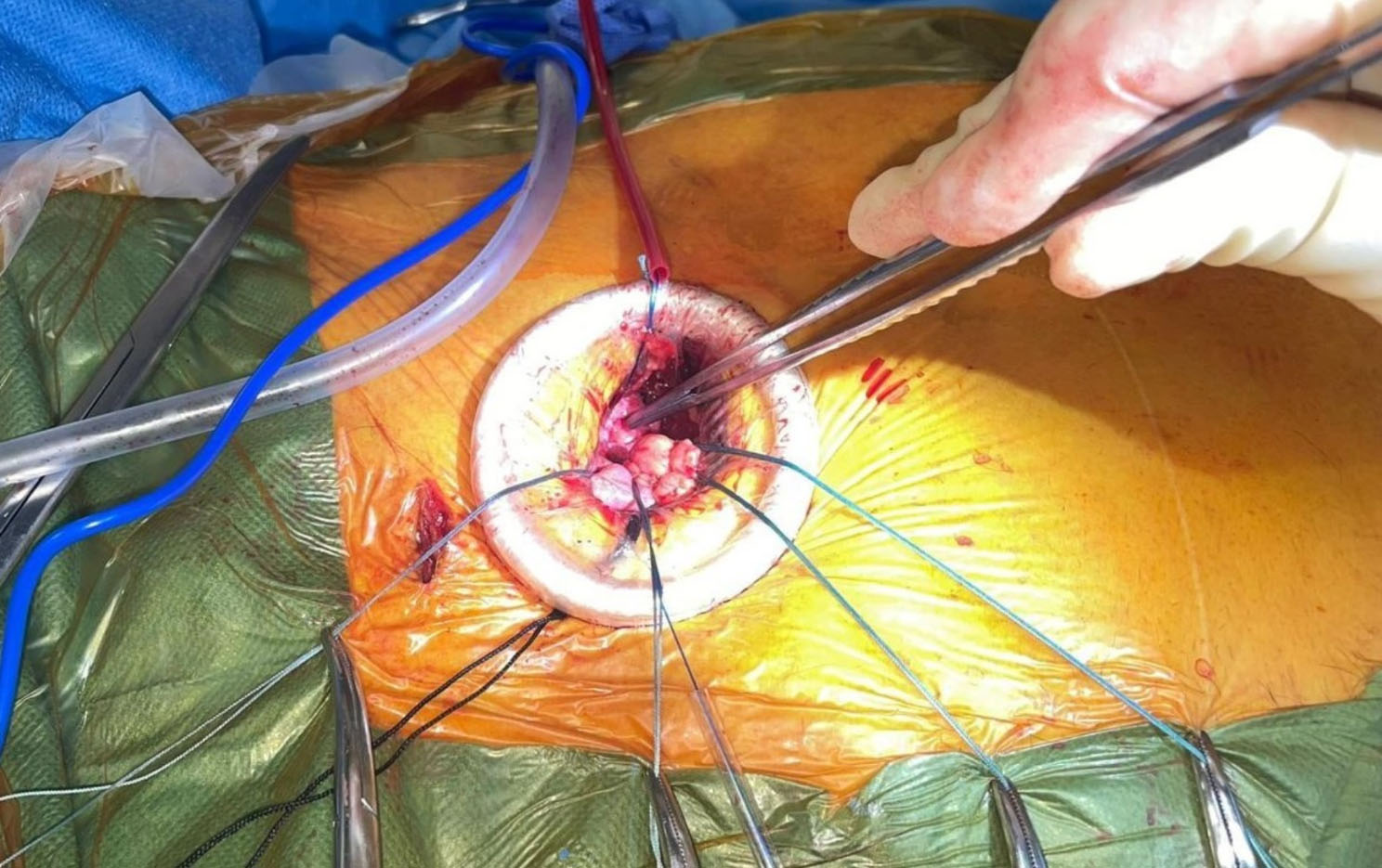
Direct aortic access site via the anterior right thoracotomy

Under general anaesthesia without using CPB, a DA TAVI via 4 cm anterior right mini-thoracotomy (ART) in the 2nd ICS (Fig. 2) was performed. A bronchial blocker for isolated lung ventilation was used. The right internal mammary artery (RIMA) was clipped and divided. Using a soft tissue retractor, the pericardium was opened and traction sutures on the pericardium inserted.

**Fig. 3:**
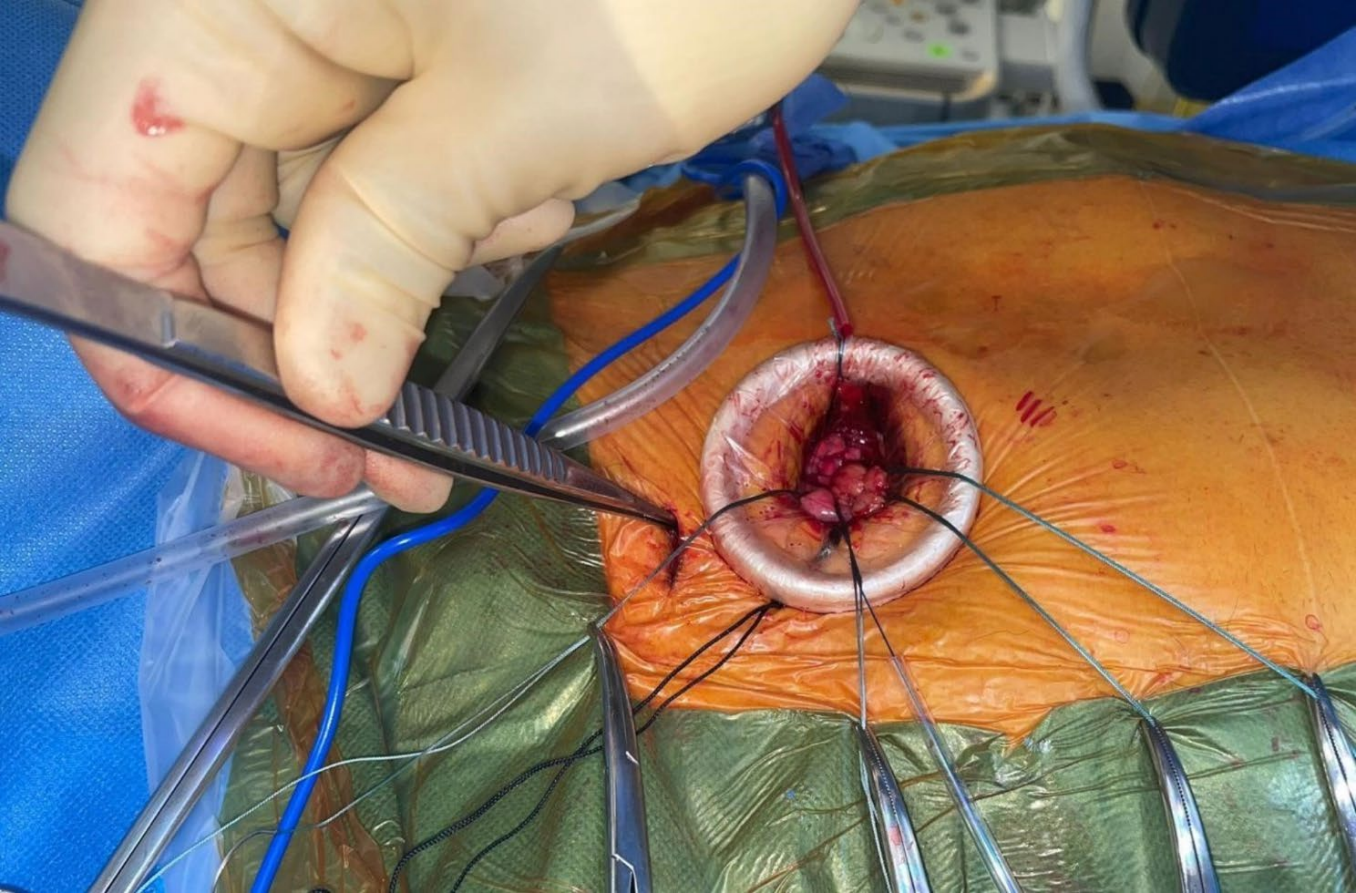
Additional small satellite puncture site above the 2nd rib

**Fig. 4:**
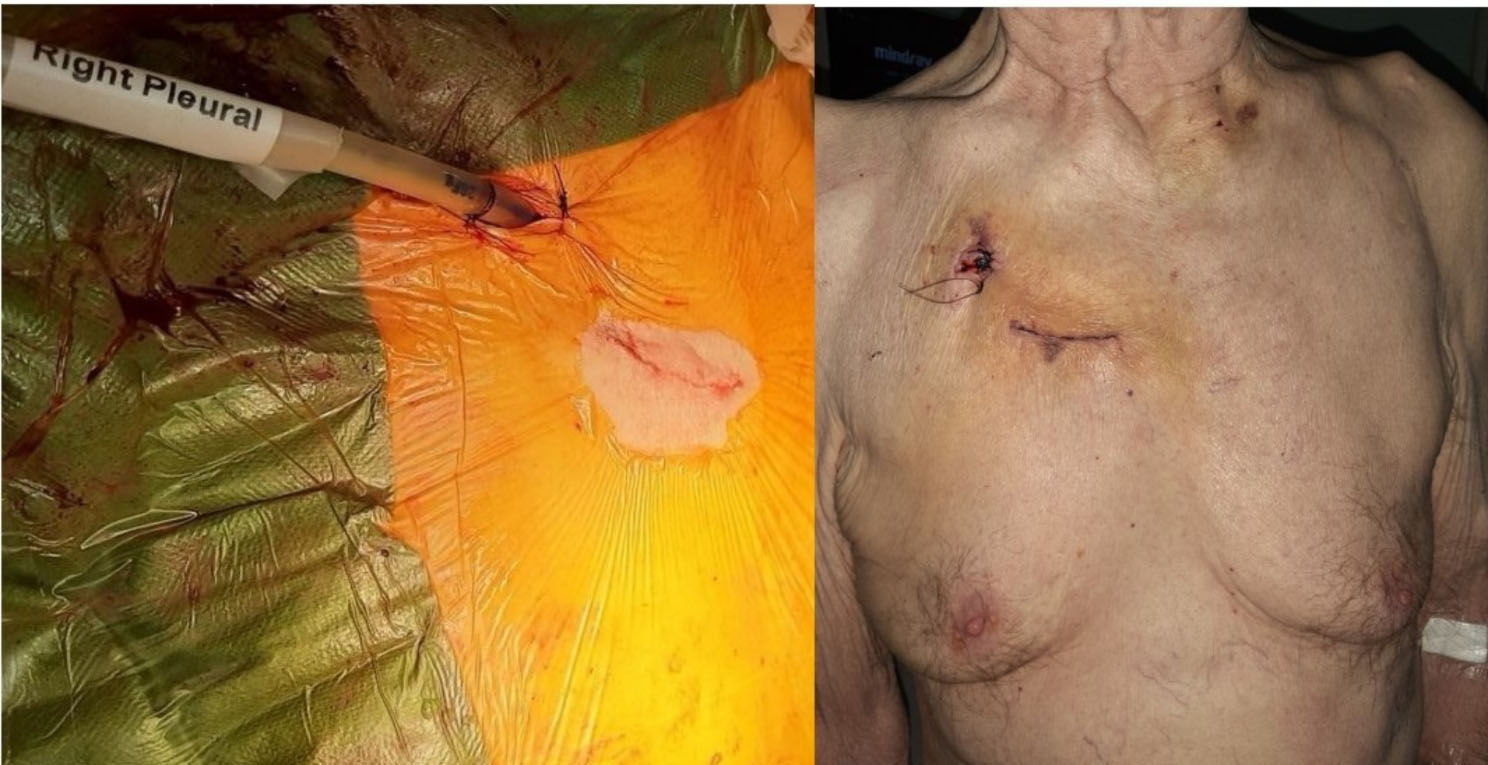
Final image of the wound appearance at the end of operation (Left) and on discharge(Right)

Arterial/venous access via the right radial artery and right femoral vein for pigtail catheter and temporary pacing wire inserted respectively. Pigtail catheter advanced into aortic root to delineate anatomy. Puncture zone identified along distal ascending aorta and 2 × 4/0 Prolene Teflon pledgeted sutures inserted. Following successful puncture via a separate access site (1st ICS) to facilitate alignment and preventing kinking of the device (Fig. 3). A 33 cm 26-Fr Gore^®^ DrySeal flex introducer sheath (GoreMedical) (Minimum I.D 8.7 mm) was delivered over a Safari Large wire into the ascending aorta. This was used instead of the 14-Fr Python™ expandable introducer sheath (Meril’s Life Sciences Pvt. Ltd) to avoid blood loss from the expanding segments. A size 32 mm MyVal was then positioned and deployed (nominal inflation) under rapid ventricular pacing. Angiogram showed no coronary obstruction with good valve positioning and no PVL (Video 2, Video 3, Video 4). TOE confirmed good functioning prosthesis and no PVL (Video 5). Aorta secured with good haemostasis and heparin reversed. The right pleura was drained via the satellite incision site.(Fig. 4). Ultrasound guided right serratus anterior block was done at the end of the procedure. Patient was extubated shortly afterwards and moved for a short period in CSICU. He was discharged home uneventfully on Day 5 postoperatively. Echo pre-discharge demonstrated good functioning aortic prosthesis with AR PHT 560 ms and VTI 0.77. Patient has been reviewed at 6 weeks with good improvement in symptoms, (Video 6).

## Discussion

Innovations of transcatheter heart valves and relating delivery systems are continuously evolving.[2] Myval-XL THV (Meril Life Sciences Pvt. Ltd., India) is a balloon-expandable valve available in larger sizes, 30.5 and 32 mm, serving to treat patients with large annulus.[3] It can protect against valve embolisation and residual PVL in non-calcified aortic valves with dilated annulus and can be a good option for treatment of pure AR in high risk patients.[4]

In instances where peripheral access is prohibitive, a trans-apical approach(through left anterior thoracotomy) or a direct aortic approach( performed through upper mini- sternotomy or anterior right mini-thoracotomy) can be considered. The ART access has been shown to reduce the risk of complications and recovery time compared to traditional surgical valve replacement.[5] In comparison to Trans-apical approach, ART is less invasive and associated with lower mortality and morbidity.[6] Additionally, using a soft tissue retractor and avoiding a rib spreader in ART results in less postoperative pain. Trans apical access was not suitable for this patient due to impaired LV function and the large size of the sheath required for the procedure. Compared to the upper mini-sternotomy approach, ART is less invasive and provides better alignment for valve implantation, although it demands a steeper learning curve for the surgeon.[7]

This patient presented with significant comorbidities and frailty, compounded by symptomatic impaired left ventricular systolic function (LVSF) .Following heart team discussion, the patient was referred for TAVI assessment. With a diagnosis of pure aortic regurgitation (AR) and an annulus measuring (Area 635mm^2^, perimeter 90.5 mm), meticulous sizing was imperative to mitigate the risk of prosthesis embolisation and a size 32 mm Myval valve (nominal implant) was deemed appropriate to provide approximately 25% oversizing. While tans-femoral TAVI is the conventional licenced approach for this valve, the patient’s history of a large saccular AAA necessitated an alternative approach. Consequently, a direct aortic approach via ART was preferred over TA access due to the larger sheath size required in addition to a moderately impaired LV. Precise guidance for the aortic puncture site under fluoroscopy was essential in addition to the pre-operative CT information. Additionally, device alignment was a critical consideration prompting the additional access site from above the ART access incision (above the 2nd rib). Following this, the TAVI procedure progressed smoothly. An effective strategy in achieving optimal valve positioning is to initially position the valve slightly deeper, facilitating subsequent adjustments upwards, rather than starting too high where re-adjustment becomes limited. In addition, DA approach allows for better proximal control of the device.

## Conclusion

This case report demonstrates the successful use of the Myval transcatheter heart valve to treat pure aortic regurgitation with a large annulus. Using a direct ascending aorta approach via ART, along with thorough pre-procedural planning and intraoperative techniques, led to favourable outcome despite the patient’s complex condition. The availability of larger valve sizes, like the size 32 mm Myval, was crucial in overcoming anatomical challenges. This emphasises the need for a tailored approach in patients with aortic valve disease and complex comorbidity. Further research and long-term follow-up are necessary to validate the effectiveness and durability of this approach.

### Electronic supplementary material

Below is the link to the electronic supplementary material.

Supplementary Material 1

Supplementary Material 2

Supplementary Material 3

Supplementary Material 4

Supplementary Material 5

Supplementary Material 6

Supplementary Material 7

## Data Availability

No datasets were generated or analysed during the current study.

## Abbreviations

TAVI: Transcatheter aortic valve implantation
ART: Anterior right mini-thoracotomy
AR: Aortic Regurgitation
THV: Transcatheter heart valve
NYHA: The New York Heart Association classification
PVD: Peripheral vascular disease
TEVAR: Thoracic endovascular aortic repair
AAA: Abdominal aortic aneurysm
COPD: Chronic obstructive pulmonary disease
LVEDD: Left ventricle end-diastolic diameter
CPB: Cardiopulmonary bypass
DA: Direct aortic
ICS: Intercostal space
RIMA: Right internal mammary artery
I.D: Internal diameter
TOE: Transoesophageal echocardiography
PVL: Paravalvular leak
SAVR: Surgical aortic valve replacement
CSICU: Cardiac surgery intensive care unit
