# Supplementary material for: Direct aortic TAVI via anterior right mini-thoracotomy using 32 mm myval for pure aortic regurgitation
Source: J Cardiothorac Surg. 2024 Aug 30;19:506. doi: 10.1186/s13019-024-02982-7 (PMC11363664; doi:10.1186/s13019-024-02982-7)
Supplement: Supplementary file 7 — Supplementary Material 7 [file 13019_2024_2982_MOESM7_ESM.docx]

**video legends:**

Video 1: Preoperative TOE images showing the eccentric severe AR.

Video 2: Access, alignment and positioning of balloon-mounted Myval THV across the aortic annulus.

Video 3: Balloon implantation of the MyVal THV under rapid pacing.

Video 4: Optimal position of the implanted valve with no paravalvular leak or coronary compromise on aortogram.

Video 5: Postoperative TOE images showing the Myval THV in situ.

Video 6: Postoperative Follow up CT scan
